# Partograph Utilization and Associated Factors among Obstetric Care Providers at Public Health Facilities in Hadiya Zone, Southern Ethiopia

**DOI:** 10.1155/2020/3943498

**Published:** 2020-04-30

**Authors:** Yosef Haile, Fikru Tafese, Tesfaye Dagne Weldemarium, Mulugeta Hailu Rad

**Affiliations:** ^1^Department of Public Health, College of Health Science, Arbaminch University, Ethiopia; ^2^Department of Health Policy and Management, Faculty of Public Health, Institute of Health, Jimma University, Ethiopia; ^3^St. Paul's Hospital Millennium Medical College, School of Public Health, Ethiopia; ^4^Department of Public Health, College of Medical and Health Science, Wachemo University, Ethiopia

## Abstract

**Background:**

A partograph is a graphic representation of labor which is used by health professionals for monitoring labor progress and fetal and maternal wellbeing. However, its utilization and associated factors have not been studied yet in Hadiya Zone, Southern Ethiopia. Hence, the aim of this study was to determine partograph utilization and associated factors among obstetric care providers at public health facilities in Hadiya Zone, Southern Ethiopia.

**Methods:**

A facility-based cross-sectional study was conducted on 436 health professionals. The study was conducted from March 04 to April 07, 2019. A simple random sampling method was carried out to select 19 health facilities and study participants from selected facilities. Data was collected using a pretested structured questionnaire, entered into EPI-data version 3.1 and exported to Statistical Package for Social Sciences (SPSS) version 20. Descriptive statistics and binary and multivariable logistic regression analyses were done. *P* values less than 0.05 were used to declare significant association between dependent and independent variables.

**Results:**

The overall magnitude of partograph utilization was found to be 54.4%, and finding from data abstraction from a document revealed that out of 18 parameters, only 10 parameters were recorded completely. Type of health facility (hospital as compared to HC) (AOR = 2.96; CI = 1.71, 5.12), received on-the-job training on partograph (AOR = 7.06; CI = 4.3, 11.37), knowledgeable about partograph (AOR = 2.12; CI = 1.3, 3.9), and favorable attitude toward partograph use (AOR = 1.8; CI = 1.12 − 2.97) were significantly associated with partograph use.

**Conclusion:**

Overall partograph utilization was low, and incomplete recording of required parameters on partograph was observed in this study. Participants who received on-the-job training on partograph, who are working in a hospital, who are knowledgeable about partograph, and who have favorable attitude toward partograph use were factors affecting partograph use positively.

## 1. Introduction

A partograph is a graphic representation of labor which is used by health professionals for monitoring labor progress and fetal and maternal wellbeing [1]. The main aim of using it is to prevent prolonged and obstructed labor which is the major cause of maternal and prenatal mortality and morbidity in developing countries [2, 3].

Partograph consists of three components, maternal and fetal condition and progress of labor. It involves various parameters to assess progress of labor and maternal and fetal conditions during labor. The progress of labor is assessed through cervical dilatation and descent of the head and uterine contractions. On the other hand, fetal condition is monitored by fetal heart rate, color of liquor, and the molding of the fetal skull. Furthermore, the maternal condition is also assessed through monitoring maternal pulse rate, blood pressure, temperature, and urine for volume, protein, and ketone bodies and an additional crucial factor in active management of labor is the timing of interventions as and when needed, such as augmentation with oxytocin, caesarean section, or transfer to a higher center [2, 4].

When partograph is used effectively, it prevents obstructed labor, which accounts for about 8% of maternal deaths worldwide and 13% maternal deaths in Ethiopia. It serves as an “early warning system” [3, 5, 6]. Even though maternal death is decreasing in the world, still it is the main health problem of low-income countries. These countries account the vast majority of total maternal death; from these, more than one-third of global maternal deaths occurred in sub-Saharan Africa alone which become 546 per 100,000 live births. In Ethiopia, maternal death is still high. According to EDHS 2016, it is estimated to be 412 per 100,000 live births. However, majority of deaths can be prevented with simple and cost-effective interventions like using partograph during labor and delivery [3, 7].

Prolonged labor and obstructed labor are the most common causes of maternal and neonatal illness and death in developing countries because of inadequacy and poor quality of obstetric care including poor utilization of partograph in monitoring of labor. The women who experience obstructed labor usually suffer from postpartum hemorrhage, uterine rupture, puerperal sepsis, and obstetric fistula. Furthermore, it is highly associated with birth trauma, birth asphyxia, stillbirths, neonatal sepsis, and neonatal deaths [3, 6, 8, 9].

It is one of the common easily preventable causes of maternal and prenatal morbidity and mortality in developing countries including Ethiopia. Thus, prevention of prolonged and obstructed labor by using a simple and inexpensive tool known as partograph is a key intervention for reduction of maternal and prenatal morbidity and mortality [10, 11].

To reduce maternal and neonatal morbidity and mortality due to obstructed and prolonged labor especially in developing countries, the World Health Organization (WHO) recommends universal and routine partograph utilization. Studies have shown that using the partograph to monitor labor can be highly effective in reducing complications from prolonged and obstructed labor for the mother and newborn [8, 12].

Despite its great importance on reducing maternal and neonatal mortality due to prolonged and obstructed labor, it is not widely used in many countries [4, 11] Studies from some developing countries have shown that the utilization of partograph is poor despite preparing the tool that is simple and inexpensive for intrapartum monitoring of labor [5, 13–15]. Likewise, some studies in Ethiopia reported low utilization of the partograph [16–18].

Studies conducted in some parts of Ethiopia showed the gap related with factors affecting partograph utilization such as type of profession, training about partograph, and the and associated factors among obstetric care providers but some factors such as preservice training on partograph and level of education were not studied well. Besides, its utilization and associated factors have not been studied yet in Hadiya Zone, Southern Ethiopia. Therefore, the aim of this study was to determine the magnitude of partograph use and identify the factors associated with its use among obstetric care providers at public health facilities in Hadiya Zone, Southern Ethiopia.

## 2. Methods and Participants

### 2.1. Study Setting and Design

A cross-sectional study was conducted in Hadiya Zone. Hadiya Zone is found in Southern Nations, Nationalities, and People's Region (SNNPR), Ethiopia. The capital city of the zone is Hossana which is about 232 km far away from the capital city (Addis Ababa). Hadiya Zone has a total area of 3542.66 square kilometers, administratively divided into 13 districts and 3 town administrations with a total population of nearly 1.69 million. According to the last year report of Hadiya Zone health department, there are 1 teaching hospital and 3 primary hospitals, 80 private clinics, 61 health centers, and 311 health post in the zone. Regarding health professionals, there are 2436 health providers; among these health workers, 1360 health workers were providing labor and delivery service. The study was conducted from March 04 to April 07, 2019.

### 2.2. Population

Randomly selected obstetric care providers who are working at selected public health facilities were included except health professionals who never attend labor cases.

### 2.3. Sample Size Determination and Sampling Procedure

#### 2.3.1. Sample Size Determination

The sample size was determined by using a single-population proportion formula, by taking prevalence of 53.85% (*P* = 0.53) which is obtained from a study conducted in Eastern Gojjam Zone, Northwest Ethiopia [19]. Consider the following assumption, 5% margin of error and 95% confidence level (*Z* = 1.96). The calculated sample size was 384. Taking nonresponse rate of 10% and design effect of 1.5 because a multistage sampling technique was employed, the total sample size was (10% × 384 + 384) = 423 × 1.5 = 635. Since the source population is less than 10,000, correction formulas were used to estimate the final sample size. Therefore, the required sample size was 436.

#### 2.3.2. Sample Size for the Qualitative Part (for Data Abstraction from a Document)

Ten recently (one week before the data collection) used tools or WHO modified partograph charts from one selected hospital and five recently used partograph charts each from 11 selected health centers were used for the document review [20].

### 2.4. Sampling Procedure

In Hadiya Zone, there are one teaching hospital, three primary hospitals, and sixty-one health centers and all of them provide labor and delivery services. A multistage sampling method was conducted to select study participants. A lottery method was carried out to select health facilities at the primary level. Thirty percent of public health facilities were included to obtain adequate sample size according to WHO recommendation. Then, the lottery method was used to select study participants after proportional allocation of health professionals found at each health facilities. To employ the lottery method on each facility, a sampling frame was prepared by listing the names of health professionals.

In the selected health centers, health professionals from all departments have a chance to be included in the study by taking into account that they are always rotated among departments including the delivery unit every two to three months. In addition, when health center staffs are assigned at night and weekend duty, they provide all types of services including delivery care. In hospitals, providers only from delivery, antenatal care (ANC), postnatal care (PNC), family planning (F/P), and gynecology units were included because the trend of rotation is only among these departments not including other departments.

#### 2.4.1. Data Collection Tools

A pretested and structured, self-administered questionnaire was adapted from previous literature [16, 18, 21–23]. The questionnaire mainly focused on sociodemographic characteristics, professional characteristics of obstetric care providers, and other factors like knowledge and attitude toward partograph utilization.

#### 2.4.2. Quantitative Data Collection Method

A pretested self-administered structured questionnaire was used for quantitative data collection. Data was collected from all selected obstetric care providers by 6 trained data collectors. Data collectors were three clinical nurses and three midwives, and training was given for them for two days. Two trained BSc midwives were also assigned for supervising and review of recently used partograph for checking the completeness of the partograph.

#### 2.4.3. Qualitative Data Collection Method

Data abstraction from recently used partograph charts was carried out by two trained midwives among ten recently used partograph charts from one hospital and five each from 11 health centers to know whether the parameters on the partograph were filled correctly and completely in an obstetric ward/unit, during active first stage of labor by using a structured checklist which is developed from the WHO modified partograph.

## 3. Operationalization

### 3.1. Partograph Utilization

The partograph is used by obstetric care givers routinely for all laboring mothers. It was measured by using two-step questions. The first step is asking the participants whether they have been using partograph or not. For the second step, the respondents who responded “yes” in the first step was required to answer “how often” they have been using partograph (occasionally, sometimes, and routinely). Finally, those who are using partograph routinely are categorized as partograph utilizers and those who never use partograph and using partograph sometimes and occasionally were considered nonutilizers. Further, partograph utilization was classified as low if partograph use in the study area was below 60% and high if it was greater than or equal to 60% [8, 16].

### 3.2. Knowledge about Partograph

Knowledge about partograph was measured by using seven knowledge-related questions. Those who scored 60% and above to knowledge-related questions were considered knowledgeable whereas those who scored less than 60% were considered not knowledgeable [24].

### 3.3. Attitude

Providers' attitude toward partograph utilization was assessed by using 5-point Likert scale questions as individuals responding strongly agree for positive attitude were given scores of 5 and 1 for those who responded as strongly disagree, while the above scores were reversed for negative attitude questions. The total score was dichotomized into favorable and an unfavorable attitude taking the mean score.

### 3.4. Obstetric Care Providers

Obstetric care providers include medical doctors, midwives, nurses, and health officers who are providing delivery service by regular time, rotation, and duty time.

### 3.5. Complete and Correct Recording of Parameters on Partograph

Complete and correct recording of parameters on partograph means recording all eighteen parameters on the space provided on the partograph and at the allowed time gap for each parameter.

#### 3.5.1. Data Processing and Analysis

Checking, coding, and organization of the collected questionnaires were done manually every day to check for completeness. The completed questionnaires were coded, and data were entered into a data entry template in EPI-data version 3.1. After checking and correcting errors, the data were exported to Statistical Package for Social Science (SPSS) version 20. The negatively worded items were reverse-coded. In the descriptive statistical analysis, frequencies, proportion, and mean were calculated and the results of the analysis were presented in texts, tables, and graphs as appropriate. Independent variables having *P* < 0.25 on binary logistic regression analysis were considered candidates for multiple logistic regression analysis.

Multiple logistic regression analysis was carried out to identify factors having statistically significant associations with partograph utilization. The final model was fitted using backward LR variable selection methods. Significant independent predictors were declared at *P* value less than 0.05, and AOR was used for interpretation.

#### 3.5.2. Ethical Consideration

The study was reviewed and approved by the IRB at the Institute of Health Sciences of Jimma University. Permission letter was obtained from the SNNPR Health Bureau and Hadiya Zone Health Department. Participants were informed about the objectives of the study, and informed consent for participation was obtained from individual participants. Finally, participants were informed that they had full right not to participate in the study.

## 4. Results

### 4.1. Sociodemographic Characteristics of Study Participants

In this study, from a total sample size, 399 participated in the study, which yielded a response rate of 91.5%. Two hundred twenty (55%) out of the total participants were female; 67.2% were between the age range of 25 and 34 (Table 1).

### 4.2. Professional Characteristics of Obstetric Care Providers

Most of the respondents were nurses by profession (44.4%) followed by midwives (35.3%) including both BSc and diploma. Regarding clinical year of experience (in years), majority of respondents (282) (70%) had clinical work experience in a range of 1-5 years. Most of the participants (276) (69.2%) were working in health centers. Out of the 399 respondents, around 227 (56.9%) of the obstetric care providers received on-the-job training on partograph and only 120 (30.1%) respondents received training on obstetric care in their health facilities (Table 2).

### 4.3. Knowledge of Obstetric Care Providers about Partograph

Two hundred sixteen (54.1%) of the obstetric care providers were knowledgeable in this study; however, almost all of the respondents (98.7%) reported that they have learned about partograph when they were in colleges/universities.

### 4.4. Attitude of Obstetric Care Providers toward Partograph Utilization

About half (52.1%) of obstetric care providers scored 3.37 or above on attitude-related questions, and they are considered obstetric providers who had favorable attitude toward partograph utilization.

### 4.5. Partograph Utilization

The overall magnitude of routine utilization of partograph among participants to monitor labor for all laboring mothers was found to be only 217 (54.4%). In addition, completeness of parameters presented on the partograph charts was also assessed by reviewing recently (one week before the data collection) used 65 partograph charts whether the components of the partograph were filled completely or not in twelve health facilities. Only ten charts were filled completely out of sixty-five recently used partograph charts. From eighteen parameters which are presented on the partograph charts, only ten parameters were filled correctly and completely. These are client name, status of membrane, fetal heart rate status of amniotic fluid, maternal blood pressure, body temperature, pulse rate, uterine contraction, parity, and initial cervical dilatation.

### 4.6. Reasons for Not Using the Partograph

**Figure 1:**
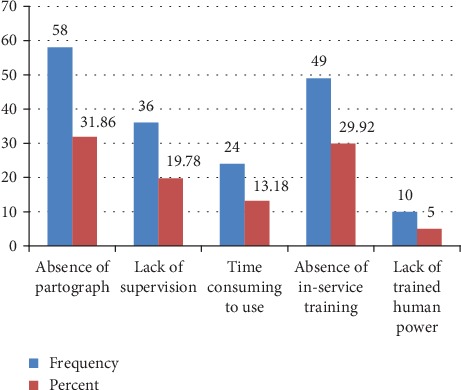
Reasons for not using the partograph by obstetric care providers of public health institution in Hadiya Zone, SNNPR Ethiopia (2019).

According to this study, from those respondents who were not using partograph routinely, the main factors that were reported by them as influencing against routine use of the partograph include unavailability of partograph in the labor ward, absence of on-the-job training, lack of supervision, using partograph is time-consuming, and lack of trained human power (Figure 1).

### 4.7. Factors Associated with Partograph Utilization by Obstetric Care Providers

#### 4.7.1. Multivariable Logistic Analysis for Factors Associated with Partograph Utilization

Out of six variables which were candidates from bivariate logistic regression analysis, four variables were significantly associated with multivariable analysis. These are type of health facility they are working, on-the-job training on partograph, knowledge about the partograph, and attitude toward partograph utilization. Those who were working in a hospital were 3 times more likely to have routine utilization of partograph than those working in health centers (AOR = 2.96 with 95% CI (1.71, 5.12)), and those health workers who were knowledgeable about partograph were about 2 times higher likely to utilize partograph routinely than those who were not knowledgeable (AOR = 2.12 with 95% CI (1.30, 3.39)). In addition, those obstetric care providers who received on-the-job training on partograph were about 7 times more likely to utilize partograph routinely than who have not received on-the-job training on partograph (AOR = 7.06 with 95% CI (4.3, 11.37)).

Furthermore, those who had favorable attitude toward partograph utilization were about 2 times more likely to utilize partograph routinely than their counterparts (AOR = 1.8 with 95% CI (1.12, 2.97)) (Table 3).

## 5. Discussion and Conclusion

In this study, only 217 (54.4%) respondents used partograph routinely for the management of labor for all laboring mothers. Type of health facility (hospitals or health centers), knowledge about partograph, on-the-job training on partograph, and attitude of participants toward partograph utilization were identified as factors which are significantly associated with partograph use.

The overall magnitude of routine utilization of partograph among participants to monitor labor for all laboring mothers was found to be only 217 (54.4%) even if using partograph is recommended for all laboring mothers by the WHO.

This finding was in line with the finding of the study conducted in Addis Ababa (57%) [25]. It is higher when compared with the finding of the studies conducted in North Shoa (40.1%) [16], Amhara Region (29%) [17], and South West Nigeria (32.2%) [8].

The possible explanation for these discrepancies might be due to the study conducted in Amhara Region which is at a regional level which includes all zones in the region; however, this thesis represents all obstetric care providers only in Hadiya Zone and in the case of North Shoa, it might be due to the difference in the number of trained obstetric care providers on partograph and the number of trained participants was higher in this study. In this study, about 56.95% participants received on-the-job training on partograph and in North Shoa, about 42.1% participants received on-the-job training on partograph. The possible explanation for the difference between the finding of the current study and South West Nigeria could be the participants of the study conducted in South West Nigeria which included HEWs in addition to other professions. This might affect partograph utilization because they may not be well trained on partograph when compared to other profession.

However, it is lower than the finding of studies conducted in Bale Zone, South West Ethiopia (73%), and Niger Delta Region of Nigeria (98.8%). The possible explanation for this difference could be the participants of the study conducted in Bale Zone had high level of knowledge about partograph which is about 61.5%; however, only 54.1% of the participants of the current study had good knowledge about partograph. The possible explanation for the difference between the current study and Niger Delta Region of Nigeria could be that the participants of the study conducted in Niger Delta Region were only midwives by profession; however, participants of the current study were from different professions who were providing obstetric care. The only 30 participating midwives on studies might increase partograph utilization because they had a great chance to be trained on partograph than other professions which might in turn improve their knowledge and skill of its utilization. Even if more than half of respondents were knowledgeable and used partograph routinely for monitoring labor, only ten parameters were filled correctly and completely from eighteen parameters which are presented in partograph charts. This implies that obstetric care providers may have a skill gap and poor attention in recording parameters in partograph charts.

According to this study, from those respondents who were not using partograph routinely, the main factors that were reported by them as influencing routine use of the partograph include unavailability of partograph in the labor ward (31.86%), absence of on-the-job training (26.92), lack of supervision (19.78%), using partograph is time-consuming (13.18%), followed by lack of trained human power (5%).

As observed from the analysis part of this study, the type of health facility the participants are working is one of the factors affecting partograph utilization. Partograph utilization was higher among those who are working in hospitals compared to those working in health centers.

This could be due to the fact that majority of obstetric care providers in hospitals were midwives and most of them were also Bachelor of Science degree holders compared to health centers, and this could increase partograph utilization because they can receive majority of obstetric training and in turn, it increases knowledge about partograph and skill of partograph utilization. It is supported by the finding of the study conducted in Bale Zone.

However, a finding observed in a study conducted in Addis Ababa revealed that those working in health centers used partograph more when compared to those who were working in hospitals. This might be because majority of the obstetric care providers working in health centers in Addis Ababa received on-the-job training on partograph and frequent supervision as compared to those working in hospitals and health centers in other places.

The other factor that was significantly associated with partograph utilization was knowledge about partograph. Those who were knowledgeable about partograph were more likely to utilize partograph than those who were not knowledgeable about partograph. The finding is consistent with findings of the studies conducted in North Shoa [16], Amhara Region [17], and Eastern Gojjam [19], and it is also supported by the findings of the studies done in Niger Delta Region of Nigeria [14] and the University of Caliber Teaching Hospital, Caliber, Nigeria [15].

In addition, on-the-job training on partograph had a significant association with partograph utilization. Those obstetric care providers who received on-the-job training on partograph were more likely to utilize partograph than those who have not received on-the-job training. This might be due to the fact that obstetric care providers who received on-the-job training had better knowledge about partograph than others that in turn improves their skills and partograph utilization. This finding is supported by the findings of the studies done in North Shoa [16], Eastern Gojjam [22], Addis Ababa [25], and Amhara Region [17].

Furthermore, it was also observed that attitude toward partograph utilization was significantly associated with partograph utilization. Those who had favorable attitude toward partograph utilization were more likely to utilize partograph when compared to those who had unfavorable attitude towards partograph utilization. This might be because those who had favorable attitude toward the use of partograph might be well motivated to improve skill and use the partograph. This finding is similar with the finding of the study conducted in North Shoa, Northern Ethiopia [16].

In conclusion, the overall partograph utilization was found to be low and incomplete recording of required parameters was observed. Type of health facility (hospitals or health centers), knowledge about partograph, training on management of labor and partograph, and attitude of participants toward partograph utilization were identified as factors which are significantly associated with partograph utilization.

### 5.1. Limitation

Some of the limitations of the study were social desirability bias and information bias (the information obtained was dependent on the participants' self-report or perception responses) as well as the study not identifying cause and effect relationship. The tool was subjected to information contamination by mood and attitude of coworkers that was controlled by informing participants to complete privately.

## Figures and Tables

**Table 1:** Sociodemographic characteristics of respondents in Hadiya Zone, SNNPR Ethiopia.

Variables		Frequency	Percent
Age category of the respondents	20-24	77	19.3
	25-29	156	39.1
	30-34	112	28.1
	≥35	54	13.5

Sex	Female	220	55.1
	Male	179	44.9

Marital status	Married	150	37.6
	Single	246	61.7
	Divorced	3	0.7

Respondent's religion	Orthodox	147	36.8
	Muslim	38	9.5
	Protestant	203	50.9
	Catholic	11	2.8

Level of education	Degree or more	259	64.9
	Diploma	140	35.1

**Table 2:** Professional characteristics of obstetric care providers in Hadiya Zone, 2019.

Variables		Frequency	Percent
Profession
	General practitioner	21	5.3
	Health officer	72	18.0
	BSc nurse	85	21.3
	Diploma nurse	80	20.1
	BSc midwives	81	20.3
	Diploma midwives	60	15.0

Type of health facility	Hospital	123	30.8
	Health center	276	69.2

Years of clinical service	1-5 years	282	70.7
	6-10 years	109	27.3
	>10 years	8	2.0

Regular working department	OPD	120	31.1
	ANC and FP	101	25.3
	Labor and delivery ward	178	44.6

Training on obstetric care	Yes	120	30.1
	No	279	69.9

Studied partograph	Yes	394	98.7
	No	5	1.3

Training on partograph	Yes	227	56.9
	No	172	43.1

**Table 3:** Multivariable analyses for factors associated with partograph utilization among obstetric care providers, Southern Ethiopia, July, 2019 (*n* = 399).

Variables	Category	Partograph utilization		AOR with 95% CI	*P* value
		Utilized frequency (%)	Not utilized frequency (%)		
Work place	Hospital	91 (26%)	32 (74.0%)	2.96 (1.71, 5.12)	0.001
	Health centers	126 (45.7%)	150 (54.3%)	1	

Training	Yes	172 (75.8%)	55 (24.2%)	7.06 (4.3, 11.37)	0.001
	No	45 (26.2%)	127 (73.8%)	1	

Knowledge	Good	143 (66.2%)	73 (33.8%)	2.12 (1.30, 3.39)	0.002
	Poor	74 (40.4%)	109 (59.6%)	1	

Attitude	Favorable	125 (60.1%)	83 (39.9%)	1.8 (1.12, 2.97)	0.015
	Unfavorable	92 (48.2%)	99 (51.8%)	1	

1 = reference group; Hosmer and Lemeshow: *P* = 0.51; classification power = 74.4%; Nagelkerke *R* square = 0.37.

## Data Availability

The [DATA TYPE] data used to support the findings of this study are available from the corresponding author upon request.
